# Memory B cell repertoire from triple vaccinees against diverse SARS-CoV-2 variants

**DOI:** 10.1038/s41586-022-04466-x

**Published:** 2022-01-28

**Authors:** Kang Wang, Zijing Jia, Linilin Bao, Lei Wang, Lei Cao, Hang Chi, Yaling Hu, Qianqian Li, Yunjiao Zhou, Yinan Jiang, Qianhui Zhu, Yongqiang Deng, Pan Liu, Nan Wang, Lin Wang, Min Liu, Yurong Li, Boling Zhu, Kaiyue Fan, Wangjun Fu, Peng Yang, Xinran Pei, Zhen Cui, Lili Qin, Pingju Ge, Jiajing Wu, Shuo Liu, Yiding Chen, Weijin Huang, Qiao Wang, Cheng-Feng Qin, Youchun Wang, Chuan Qin, Xiangxi Wang

**Affiliations:** 1 CAS Key Laboratory of Infection and Immunity, National Laboratory of Macromolecules, Institute of Biophysics, Chinese Academy of Sciences, Beijing, China; 2 University of Chinese Academy of Sciences, Beijing, China; 3 Key Laboratory of Human Disease Comparative Medicine, Chinese Ministry of Health, Beijing Key Laboratory for Animal Models of Emerging and Remerging Infectious Diseases, Institute of Laboratory Animal Science, Chinese Academy of Medical Sciences and Comparative Medicine Center, Peking Union Medical College, Beijing, China; 4 State Key Laboratory of Pathogen and Biosecurity, Beijing Institute of Microbiology and Epidemiology AMMS, Beijing, China; 5 Sinovac Biotech, Beijing, China; 6 Division of HIV/AIDS and Sex-Transmitted Virus Vaccines, Institute for Biological Product Control, National Institutes for Food and Drug Control (NIFDC), Beijing, China; 7 Key Laboratory of Medical Molecular Virology (MOE/NHC/CAMS), Shanghai Institute of Infectious Disease and Biosecurity, Shanghai Frontiers Science Center of Pathogenic Microbes and Infection, School of Basic Medical Sciences, Shanghai Medical College, Fudan University, Shanghai, China; 8 Acrobiosystems, Beijing, China

**Keywords:** Microbiology, SARS-CoV-2

## Abstract

Omicron (B.1.1.529), the most heavily mutated SARS-CoV-2 variant so far, is highly resistant to neutralizing antibodies, raising concerns about the effectiveness of antibody therapies and vaccines^1,2^. Here we examined whether sera from individuals who received two or three doses of inactivated SARS-CoV-2 vaccine could neutralize authentic Omicron. The seroconversion rates of neutralizing antibodies were 3.3% (2 out of 60) and 95% (57 out of 60) for individuals who had received 2 and 3 doses of vaccine, respectively. For recipients of three vaccine doses, the geometric mean neutralization antibody titre for Omicron was 16.5-fold lower than for the ancestral virus (254). We isolated 323 human monoclonal antibodies derived from memory B cells in triple vaccinees, half of which recognized the receptor-binding domain, and showed that a subset (24 out of 163) potently neutralized all SARS-CoV-2 variants of concern, including Omicron. Therapeutic treatments with representative broadly neutralizing monoclonal antibodies were highly protective against infection of mice with SARS-CoV-2 Beta (B.1.351) and Omicron. Atomic structures of the Omicron spike protein in complex with three classes of antibodies that were active against all five variants of concern defined the binding and neutralizing determinants and revealed a key antibody escape site, G446S, that confers greater resistance to a class of antibodies that bind on the right shoulder of the receptor-binding domain by altering local conformation at the binding interface. Our results rationalize the use of three-dose immunization regimens and suggest that the fundamental epitopes revealed by these broadly ultrapotent antibodies are rational targets for a universal sarbecovirus vaccine.

## Abstract (web summary)

Individual antibodies identified in the blood of people triple-vaccinated against SARS-CoV-2 predominantly bind spike protein and are highly effective at neutralizing SARS-CoV-2 variants, including Omicron (B.1.1.529).

## Main

**Fig. 1:**
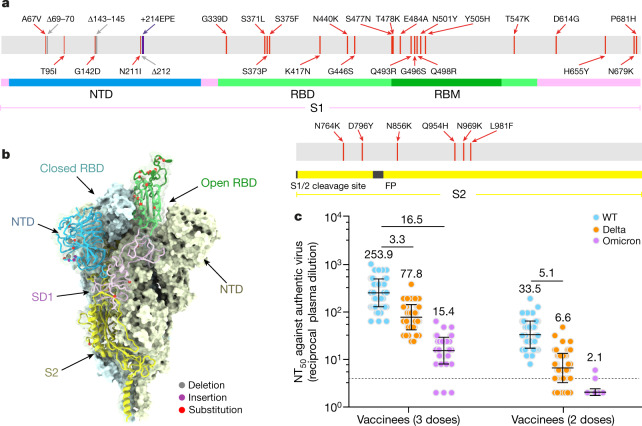
Evolution and neutralization characteristics of Omicron variant. **a**, A linear representation of Omicron S with mutations indicated. The replacements are in red, deletions are in grey and insertions are in purple. **b**, Distribution of Omicron S mutations on the cryo-EM structure^34^ of pre-fusion S trimer determined at pH 7.5 (Protein Data Bank (PDB) ID 7WG6). The mutations listed in **a** are indicated in the ‘up’ protomer shown in cartoon, with mutated residues highlighted as spheres and coloured as in **a**. The RBD, NTD, SD1 and S2 domains of this subunit are marked with arrows and coloured green, blue, magenta and yellow, respectively; the other two protomers are in the ‘down’ state and shown in surface representation in pale cyan and pale yellow. Alpha, B.1.1.7; Gamma, P.1; Lambda, C.37. **c**, The neutralizing antibody response against WT and Omicron SARS-CoV-2 authentic virus for sera from healthy vaccinees who received two (*n* = 60 volunteers) or three (*n* = 60 volunteers) doses of Coronavac. Data are geometric mean ± s.d. of technical triplicates. The dotted line represents the detection limit. NT_50_ values of less than 4 were plotted as 2. Fold difference in neutralizing antibody titre Delta or Omicron over WT for each group of sera is shown above each set of points.

The ongoing evolution and emergence of SARS-CoV-2 variants has increased concerns about the effectiveness of monoclonal antibody therapies and vaccines^3–5^, posing challenges for global pandemic control. These variants have been classed as variants of interest (VOI) or variants of concern (VOC) by the World Health Organization (WHO). The more recently identified Omicron variant, designated as a new VOC, has led to a surge in COVID-19 cases in South Africa and is now spreading across the world^6^. Omicron is the most heavily mutated variant to emerge so far, with more than 30 mutations in its spike (S) protein, 15 of which occur in the receptor binding domain (RBD). In addition, there are three small deletions and one three-residue insertion in the N-terminal domain (NTD) of the S1 subunit (Fig. 1a). The pattern of some of these alterations, similar to the those noted in previous VOCs, such as Δ69–70 in Alpha (B.1.1.7), N501Y in Alpha, Beta and Gamma (P.1), and P681H in Alpha and Delta (B.1.617.2), are associated with enhanced transmissibility, whereas many substitutions, including G142D/Δ143–145, ins214EPE, K417N, T478K, E484A, Q493R and N501Y, are closely linked with resistance to neutralizing antibodies and vaccine induced humoral immunity^3,5,7–11^ (Fig. 1a, b).

Although COVID-19 vaccines have continued to be effective against severe diseases and deaths, including those caused by the circulating Delta variant, waning immunity and massive breakthrough infections caused by viral diversification warrant a third vaccine dose or new vaccines. To combat the current resurgence of the epidemic, the US Food and Drug Administration has authorized use of a third booster dose for all adults after completion of primary vaccination with approved COVID-19 vaccine^12^. This step seems essential, because preliminary studies have indicated that three doses of Pfizer-BioNtech mRNA vaccine neutralize the Omicron variant with an approximately 40-fold decline in viral titre, whereas two doses are less effective^1,13^. However, these preliminary data on the sensitivity of Omicron to neutralization require further independent confirmation. The clinical effects of natural and vaccine-induced immunity in relation to protection from infection and severe disease require urgent investigation.

## Authentic Omicron neutralization

CoronaVac, a β-propiolactone-inactivated SARS-CoV-2 vaccine against COVID-19, has been approved for emergency use and recommended for a booster (third) vaccination dose in older people by the WHO^14,15^. We collected serum specimens from two groups of individuals who had received two doses (*n* = 60, at *t* = 0 and 1 month) or three doses (*n* = 60, at *t* = 0, 1 and 7 months) of CoronaVac to evaluate neutralization titres against the Omicron and Delta variants using live SARS-CoV-2. None of the volunteers recruited for vaccination were infected with SARS-CoV-2 before the study. Blood samples were collected from vaccinees four weeks after their last vaccination to compare neutralizing antibody titres against circulating SARS-CoV-2 variants. We used an early passage of isolated (CHK06 strain) and sequence-confirmed live Omicron virus for neutralization assays in this study. Among recipients of three doses of CoronaVac, the geometric mean half-maximal neutralizing titres (GMT NT_50_) against live wild-type (WT) virus, Delta and Omicron variants were 253.9, 77.8 and 15.4, respectively. Compared with WT virus, neutralizing titres against Delta and Omicron were, on average, 3.3-fold and 16.5-fold lower, respectively (Fig. 1c). Only 3 out of 60 samples had a NT_50_ titre of less than 8 against Omicron, with a seroconversion rate of 95% for neutralizing antibodies (Fig. 1c). However, the effectiveness of a two-dose vaccine regime against Omicron infection is relatively low. Among recipients of two doses of CoronaVac, the mean NT_50_ titre against Delta was 6.6, 5.1-fold lower compared with WT virus, but none of the serum specimens had an NT_50_ titre higher than 8 against Omicron (Fig. 1c). Compared with vaccinees who had received two doses, sera from vaccinees who had received three doses exhibited smaller reductions in neutralization titres against Delta, consistent with previous observations that three-dose administration of inactivated virus vaccine leads to enhanced neutralizing breadth against SARS-CoV-2 variants^7^.

## Antibodies elicited by three-dose vaccination

**Fig. 2:**
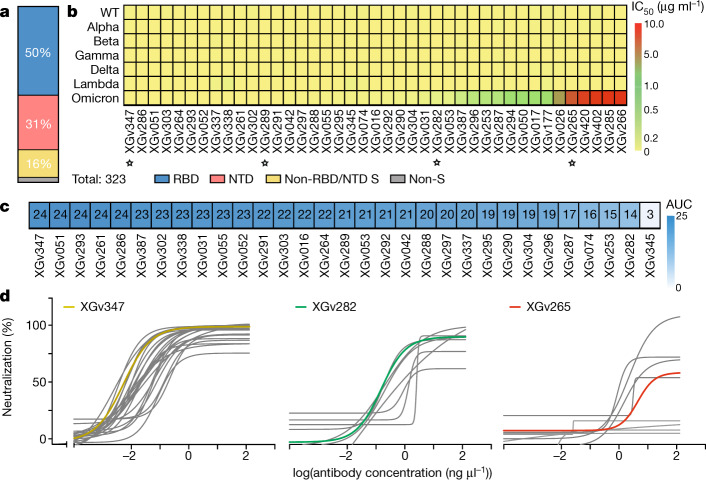
Characteristics of a subset of broadly neutralizing antibodies from recipients of a booster immunization. **a**, Vertical slice chart shows the gross distribution of binding epitopes of monoclonal antibodies isolated from individuals who received three doses of inactivated SARS-CoV-2 vaccine. The total number of antibodies and the percentage of antibodies that recognize the RBD, NTD and S2 domain are indicated. **b**, Heat map representation of 41 selected representative monoclonal antibodies against pseudotyped viruses expressing WT or variant SARS-CoV-2 S. The colour bar on the right shows IC_50_ values for the indicated monoclonal antibodies against pseudotyped viruses in **c**. Antibodies marked with star were selected for structural analysis. **c**, Heat map showing the competition ability of selected monoclonal antibodies with human ACE2. Competition ability is represented by the AUC, ranging from 1 (weakest) to 24 (strongest). **d**, Neutralization curves for the selected antibodies towards pseudotyped viruses expressing Omicron S. Data represent three groups of antibodies shown in **b**. Yellow indicates antibodies with high neutralizing activity against all five VOCs; green indicates antibodies with high neutralizing activity against four VOCs and intermediate neutralizing activity against Omicron; red indicates antibodies with high neutralizing activity against four VOCs and weak neutralizing activity against Omicron. XGv347, XGv282 and XGv265 were selected as representatives of each group. All experiments were performed in duplicate.

We previously sorted immunoglobulin-expressing (IgG+) memory B cells from peripheral blood mononuclear cells (PBMCs) of four individuals who had received three doses of CoronaVac using prefusion SARS-CoV-2 S as a bait^7,16^. In total, we sorted 1,800 SARS-CoV-2 S-specific memory B cells, obtained 422 paired heavy chain and light chain antibody sequences, and selected 323 antibodies for expression (Supplementary Table 1). Characterization by enzyme-linked immunosorbent assay (ELISA) showed that 163, 100 and 51 antibodies recognized the RBD, NTD and S2 domain, respectively and 9 antibodies did not bind S (Fig. 2a). Affinity measurements using biolayer interferometry (BLI) showed that nearly all RBD-directed antibodies bound to WT SARS-CoV-2 S at sub-nanomolar levels (Supplementary Table 1), and we selected 127 of these antibodies showing neutralization activities against both authentic and pseudotyped WT SARS-CoV-2 for further investigation. More than 93% of these antibodies exhibited broad binding activities to most VOCs and VOIs (Supplementary Table 1). Notably, 85% of these antibodies cross-reacted with the Omicron S RBD (Supplementary Table 1). Around 80% of the antibodies that bound the NTD did not bind Omicron S. Additionally, NTD antibodies also showed relatively poor cross-reactivity to S from the other four VOCs, owing to the increased diversity of the NTD compared with other regions of S (Fig. 1a, b, Supplementary Table 1).

## Monoclonal antibodies with broad neutralization

**Extended Data Fig. 1:**
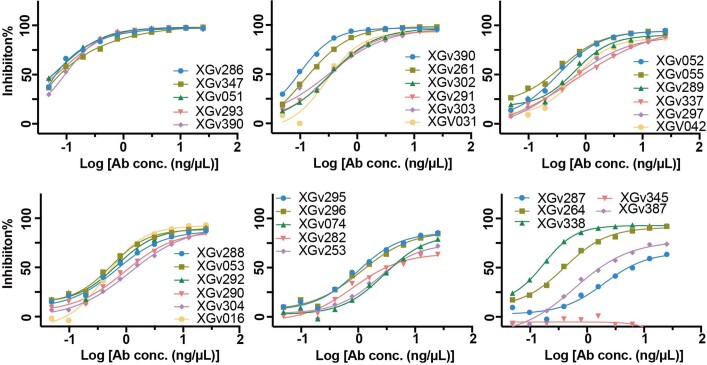
Antibody-hACE2 competition ELISA assay. Data shown are the curves of 31 antibodies used to compete with ACE2. All experiments were performed in duplicate.

**Extended Data Fig. 2:**
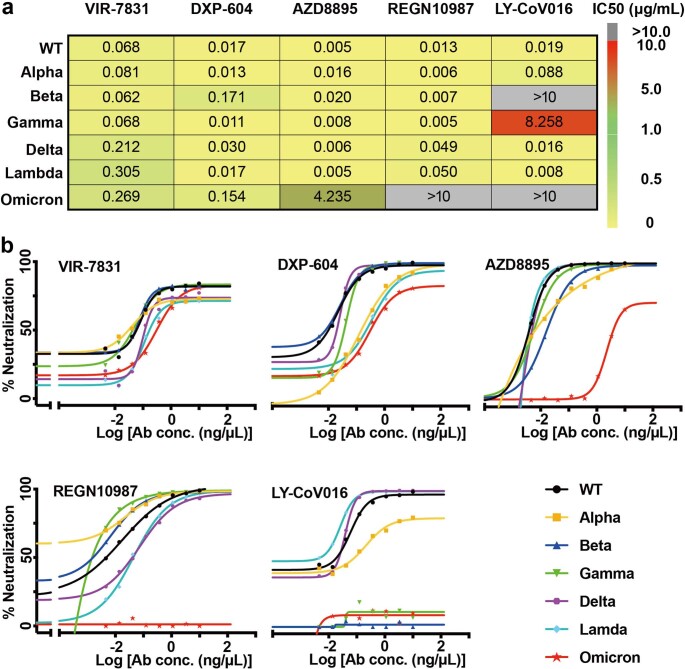
Characteristics of representative antibodies against pseudotyped viruses. **a**, Heatmap representation of five therapeutic mAbs approved or in clinical trials against pseudotyped viruses with the S proteins of wild-type or variants of concern or interst (Alpha, Beta, Gamma, Delta, Lambda and Omicron). **b**, Neutralization curves for these mAbs in correspondence with **a**. Mean of two experiments is shown.

**Extended Data Fig. 3:**
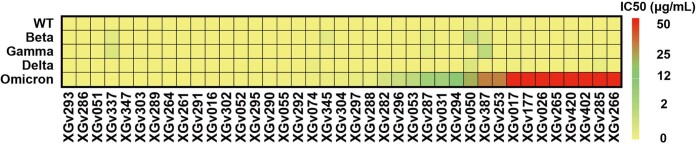
Heatmap representation of representative mAbs against WT and variants of concern. Color bar on the right showed the gradient of IC50 of different antibodies against the authentic WT and variants of concern. All experiments were performed in duplicate.

Results of pseudovirus neutralization assays performed using virus expressing the S of WT virus or VOCs^17,18^ identified 31 RBD-targeting antibodies that were particularly potent, with half-maximal inhibitory concentration (IC_50_) values ranging from 0.002 to 0.800 μg ml^−1^ against WT virus and the VOCs (Fig. 2b). Among these, 30 antibodies neutralized the virus by directly blocking the interactions between the RBD and its receptor, human angiotensin-converting enzyme 2 (ACE2), and 1 antibody used other mechanisms to neutralize viral infection (Fig. 2c, Extended Data Fig. 1). A subset of RBD antibodies (13 and 24) neutralized virus expressing Omicron S, with IC_50_ values below 0.02 μg ml^−1^ and 0.1 μg ml^−1^, respectively. This neutralization is as potent as neutralization by best-in-class antibodies against virus expressing WT S (Fig. 2b, d, Supplementary Table 1, 2)—we obtained IC_50_ values of 0.27 and 0.16 μg ml^−1^ for the well-studied therapeutic antibodies VIR-7831 and DXP-604, respectively. These values are 10- to 40-fold higher than those of the subset antibodies (Extended Data Fig. 2, Supplementary Table 1). Neutralization activity of some antibody drugs, such as REGN10933, REGN10987, LY-CoV555, LY-CoV016, AZD1061 and AZD8895, was almost completely lost with virus expressing Omicron S^2^ (Extended Data Fig. 2, Supplementary Table 1). Meanwhile, specific antibodies with high neutralizing potency against WT and some VOCs (IC_50_ < 0.2 μg ml^−1^) were identified and these comprised approximately 30% of the antibody repertoire (Supplementary Data Table 1). A previous study revealed that the numbers of nucleotide mutations in the V gene for RBD-specific antibodies in individuals who had received three doses of SARS-CoV-2 vaccine were substantially higher than in individuals who had received two doses, and antibodies from individuals who had received three doses exhibited higher binding activities than those from individuals who had received two doses^5^—these results indicated the evolution of a wide range of antibodies over time. Experiments repeated using authentic virus, including WT virus and five circulating VOCs, showed similar neutralization patterns by all these antibodies (Extended Data Fig. 3), further verifying the neutralizing potency and breadth for this subset of antibody repertoire elicited by three doses of vaccine.

## Structures of Omicron S trimer and antibodies

**Fig. 3:**
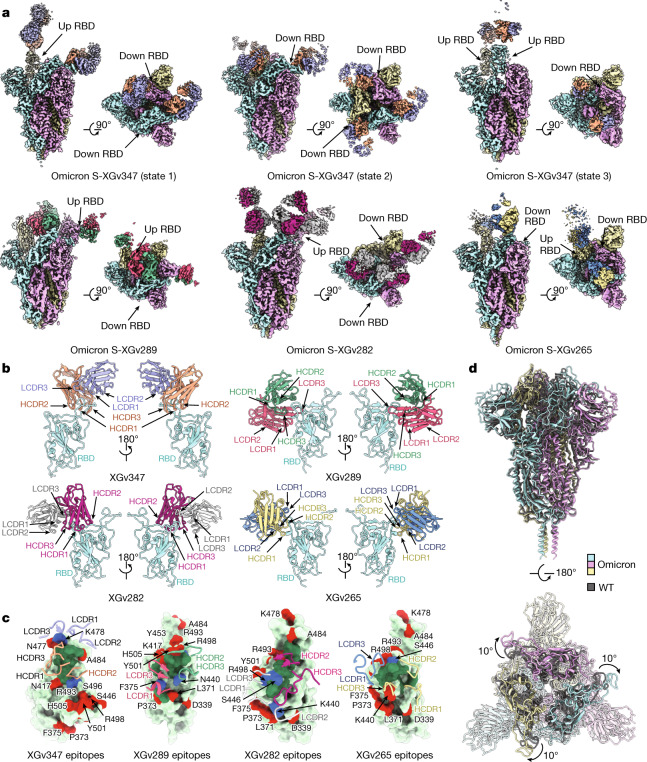
Structural basis of the broad and potent neutralization of representative antibodies. **a**, Side and top views of Cryo-EM maps of Omicron S trimer in complex with XGv347 (S states 1–3), XGv289, XGv282 and XGv265. State 1, one up RBD and one down RBD; state 2, three down RBDs; state 3, two up RBDs. **b**, Cartoon representations of the structures of Omicron S RBD in complex with XGv347 (top left), XGv289 (top right), XGv282 (bottom left) and XGv265 (bottom right). Two views are shown to illustrate the binding modes of the four antibodies. RBD is shown in cyan. **c**, Interactions between the four antibodies and Omicron S RBD. The CDRs of the four antibodies that interact with the RBD are shown as cartoon over the light green surface of RBD. The mutation sites on Omicron S RBD are in red; the epitopes of antibodies are in deep green and the overlap of mutation sites and epitopes are in blue. Residues of each epitope are indicated in the corresponding regions. **d**, Superposition of Omicron and WT S trimers. Omicron S trimer is in cyan and WT S trimer is in yellow.

**Extended Data Fig. 4:**
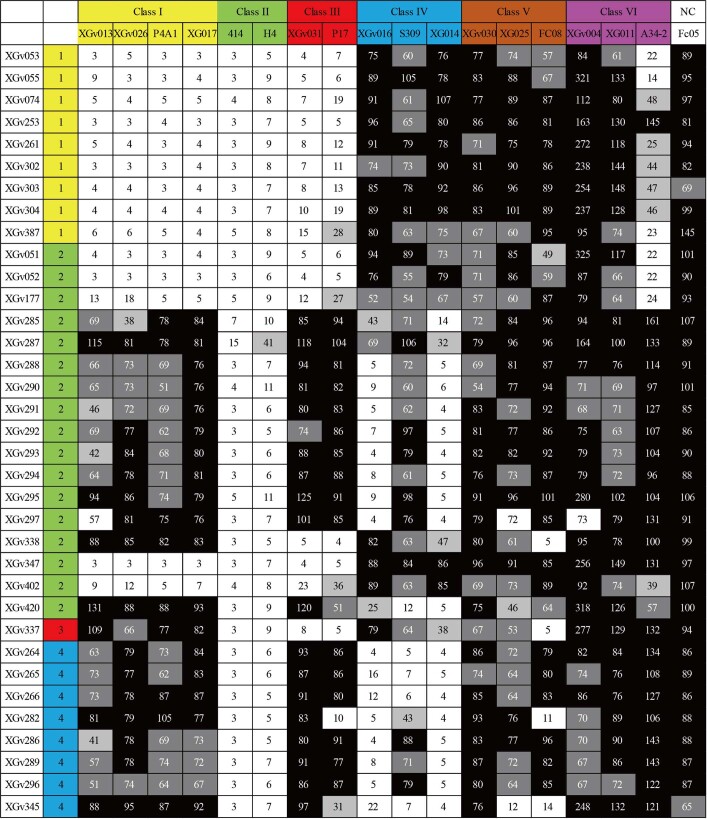
Data sheets of ELISA assay of representative mAbs against Omicron RBD. Different Classes of mAbs (Class I-VI) are colored by yellow, green, red, blue, brown and magenta, respectively. Values are filled with black (>75), grey (50–75), silver (25–50) and white (<25). Each data is the mean of three values from three independent experiments.

**Extended Data Fig. 5:**
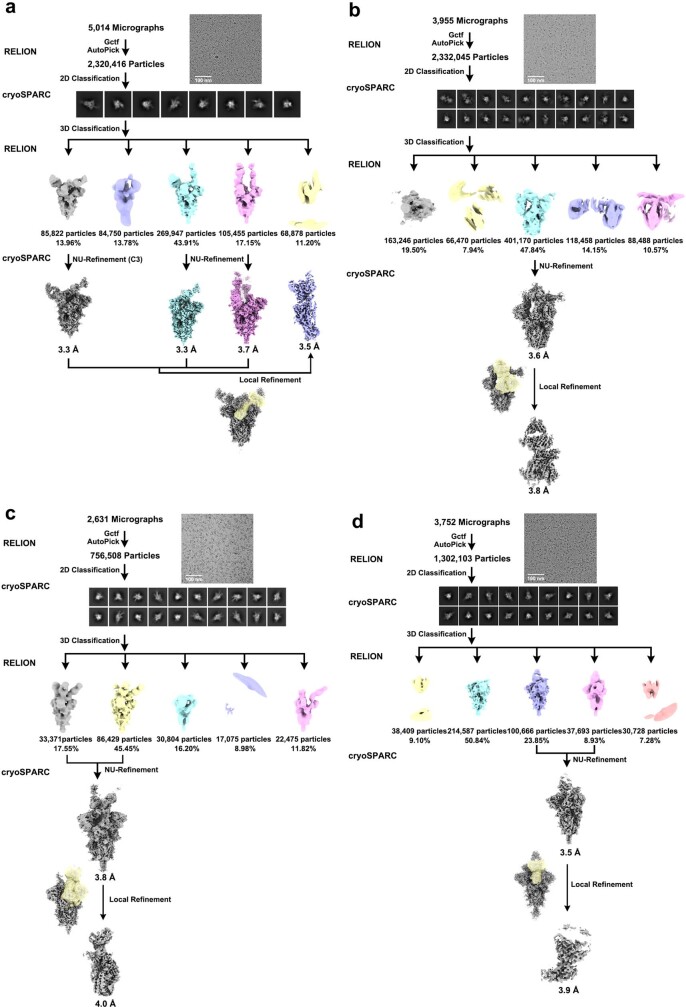
Flowcharts for cryo-EM data processing. Flowcharts for Omicron S protein in complex with **a**, XGv347, **b**, XGv289, **c**, XGv282 and **d**, XGv265 are shown. Scala bar in micrographs, 100 nm.

**Extended Data Fig. 6:**
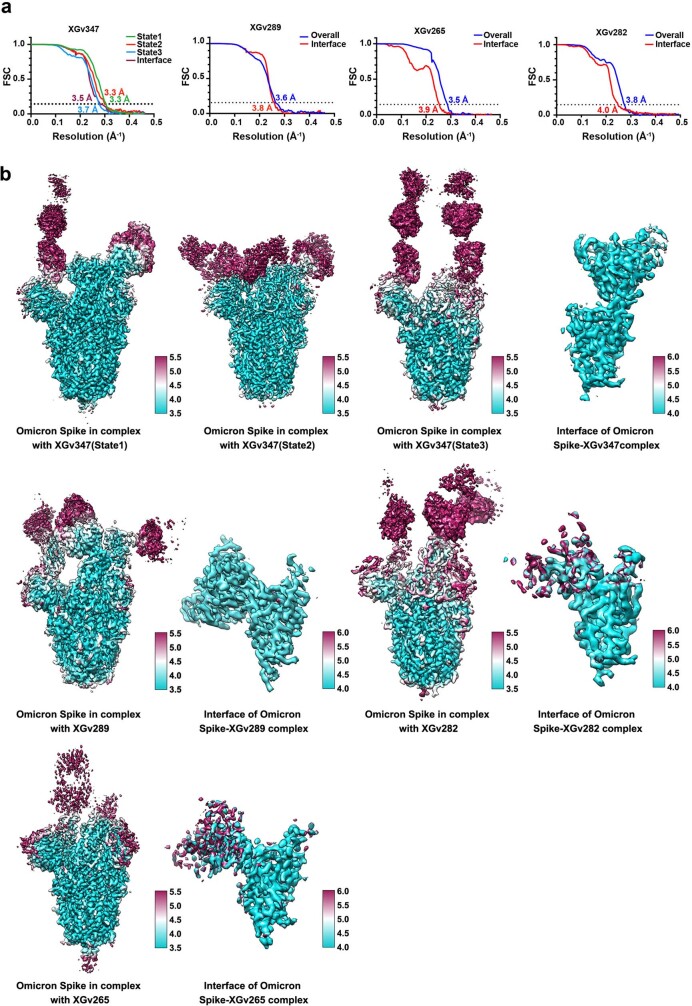
Resolution estimation of the EM maps. **a**, The gold-standard FSC curves of overall maps of Omicron S trimer in complex with Fab XGv347, XGv289, XGv282 and XGv265 and local maps of interfaces. **b**, Local resolution assessments of cryo-EM maps using ResMap are shown.

**Extended Data Fig. 7:**
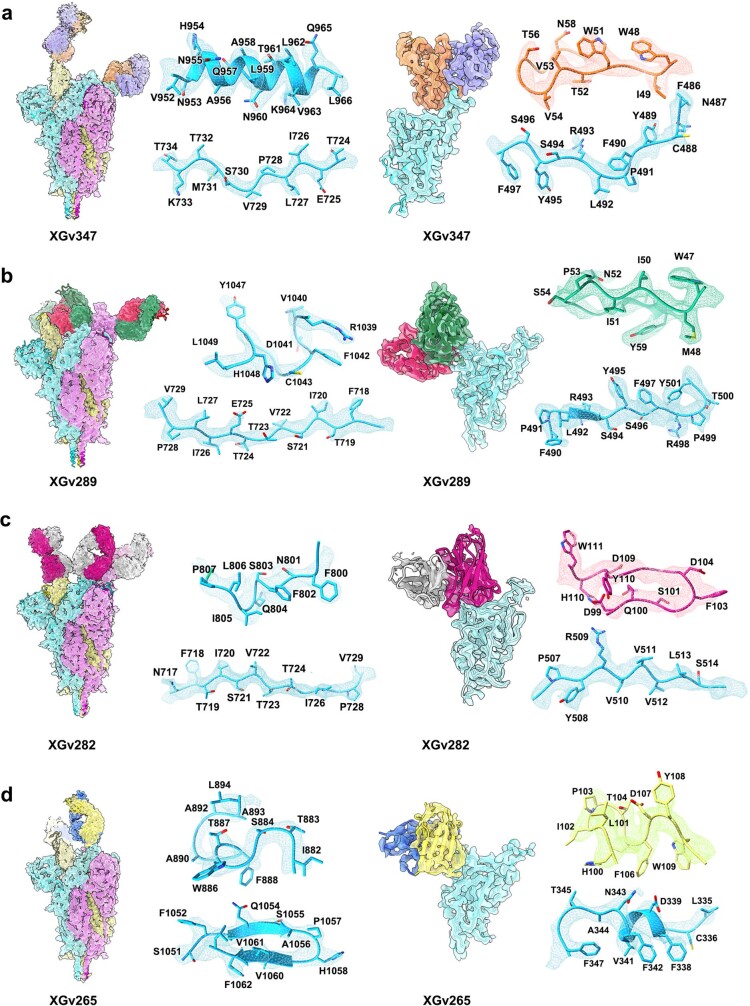
Density maps and atomic models. Cryo-EM density maps of Omicron S trimer in complex with XGv347, XGv289, XGv282 and XGv265 and their interfaces are shown. Color scheme is the same as in Fig. 3a. Residues are shown as sticks with oxygen colored in red, nitrogen colored in blue and sulfurs colored in yellow.

Antibodies targeting the RBD can be categorized into six general classes (I–VI) on the basis of cluster analysis of epitopes of 265 available RBD–neutralizing antibody complex structures^7^—these classes are related to the previously reported four classes on the basis of competition with the ACE2 for binding to S and recognition of the ‘up’ or ‘down’ states of the three RBDs in S^19–21^. ELISA-based square competition-matrix analysis with the aid of existing structural data revealed the presence of three major groups in this subset of antibody repertoire (Extended Data Fig. 4). To delineate the structural basis for antibody-mediated neutralization, we determined the cryo-electron microscopy (cryo-EM) structure of a prefusion-stabilized Omicron S trimer in complex with representative Fab fragments. The two highly potent antibodies against Omicron (XGv347 and XGv289, with IC_50_ values of 0.006 and 0.016 μg ml^−1^, respectively), one monoclonal antibody (XGv282 with IC_50_ of 0.268 μg ml^−1^) with intermediate neutralizing activities against Omicron, but high neutralizing activities against the other four VOCs, and a monoclonal antibody (XGv265 with IC_50_ of 7.479 μg ml^−1^) with more than 500-fold decreased neutralization against Omicron, but potent neutralization against four other VOCs were selected for structural investigations (Fig. 2b). We obtained cryo-EM reconstructions of these complexes at 3.3–3.8 Å, and performed local refinement to further improve the densities around the binding interface between RBD and antibodies, enabling reliable analysis of the details of the interaction (Fig. 3, Extended Data Figs. 5–7, Extended Data Table 1).

**Extended Data Fig. 8:**
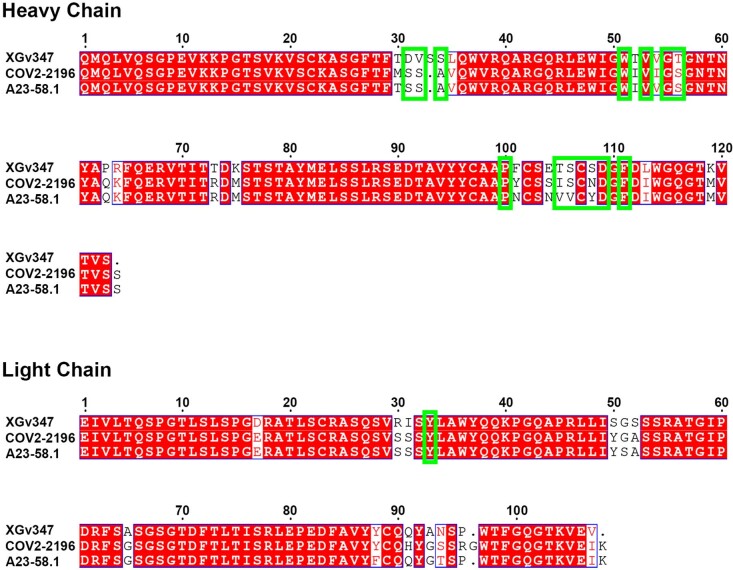
Multiple sequence alignment of XGv347, CoV2-2196 and A23-58.1. Multiple sequence alignments of heavy chains and light chains of XGv347, CoV2-2196 and A23-58.1 were performed, respectively. Paratopes of XGv347 binding to Omicron variant RBD are highlighted by green boxes.

**Extended Data Fig. 9:**
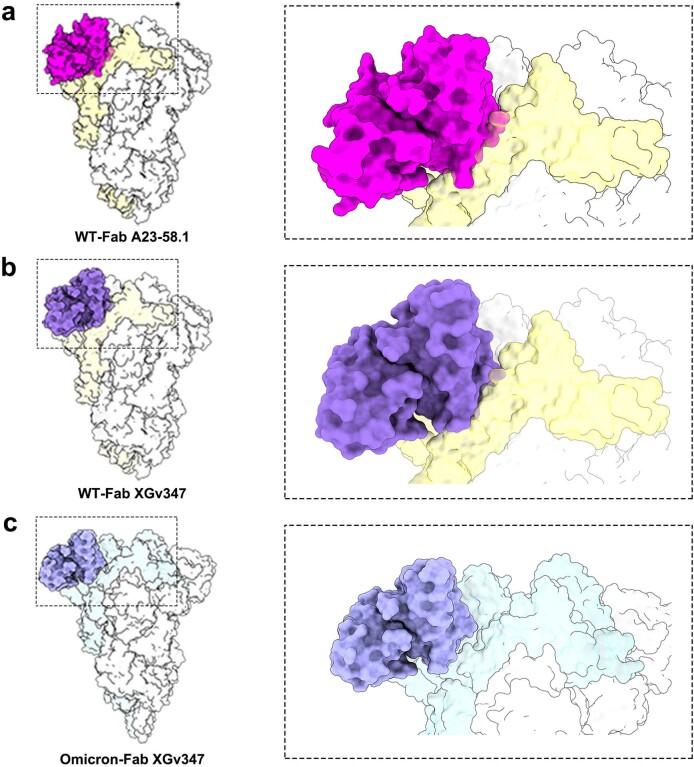
Mechanism of XGv347 binding to 3 closed RBD. **a**, Superimposition of A23-58.1 onto WT S trimer. **b**, Superimposition of XGv347 onto WT S trimer. **c**, complex of XGv347 and Omicron S trimer. All complexes are in the same orientation with close-ups of Fab-RBD binding modes showing potential clashes.

**Extended Data Fig. 10:**
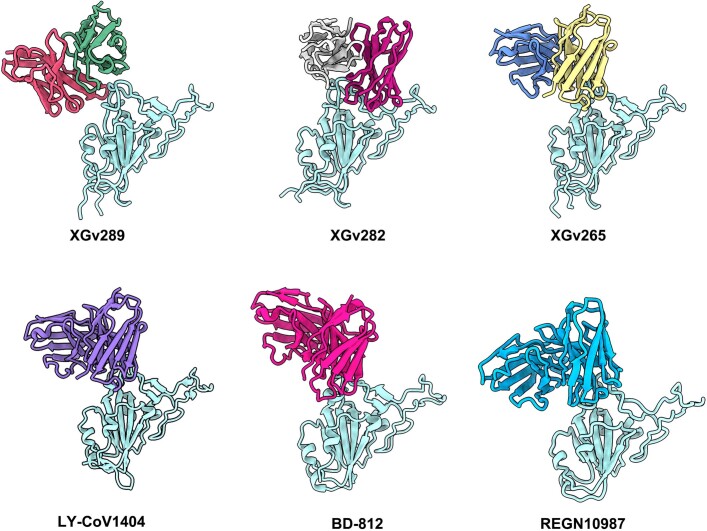
Binding modes of XGv289, 282 and 265. Binding modes of XGv289, XGv282 and XGv265. RBD is colored in light cyan and color scheme of XGv289, XGv282 and XGv265 is the same as in Fig. 3a. LY-CoV1404, BD-812 and REGN10987 are colored in purple, deep pink and blue, respectively.

**Extended Data Fig. 11:**
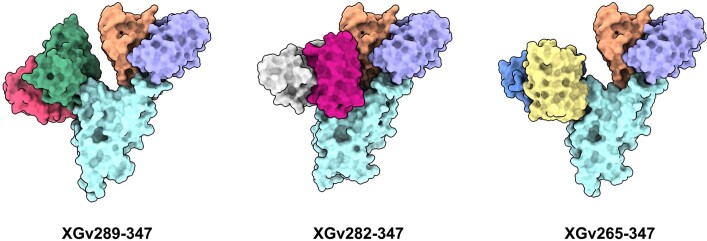
Structural fitting. XGv265, XGv282 and XGv289 are superimposed onto XGv347 and all structure are shown as surface.

**Extended Data Fig. 12:**
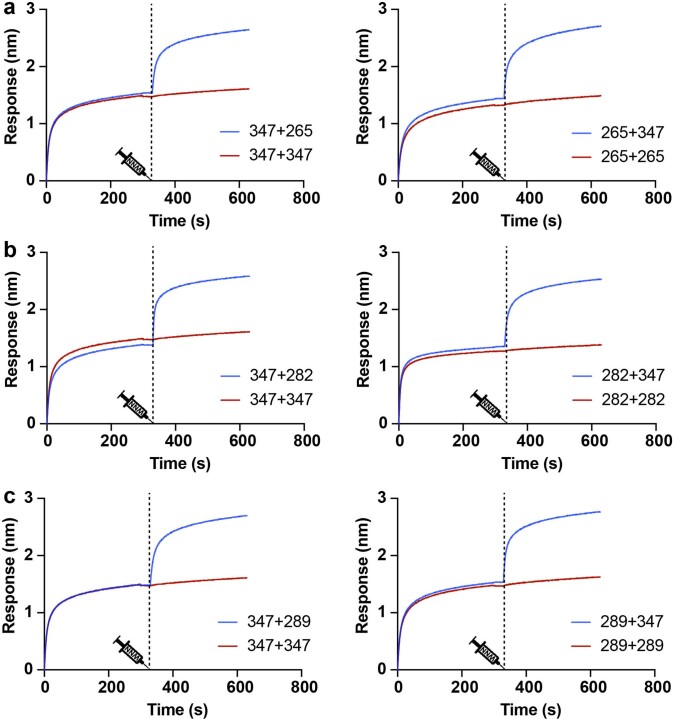
BLI assay for XGv347 competing with XGv289, XGv282 and XGv265. Affinity curves of XGv347 to Omicron S protein competing with **a**, XGv265, **b**, XGv282 and **c**, XGv289. In each panel, (left) XGv347 was first injected, followed by the XGv265, XGv282 and XGv289 in **a**–**c**, respectively. (right) Also, XGv265 in **a**, XGv282 in **b** and XGv289 in **c**, was injected first and competed with the second injection of XGv347. Each curve is a representative of three independent experiments.

The strauctures of XGv347–Omicron S structures revealed three distinct conformational states: three XGv347 Fabs bound to a completely closed S with three RBDs in the down state; two XGv347 Fabs bound to RBD in either two up or one up and one down configuration (Fig. 3a). By contrast, each of the complex structures for XGv289, XGv282 and XGv265 showed only one configuration, in which three XGv289 Fabs bound to two up and one down RBDs; three XGv282 Fabs bound to one up and two down RBDs; two XGv265 Fabs bound to an S trimer with one down and one up RBD, although the XGv265-bound up RBD conformation was weakly resolved and therefore not modelled (Fig. 3a). XGv347 bound to an epitope at the tip of the RBD, largely overlapping with the patch targeted by ACE2 (Figs. 2c, 3b, c, Extended Data Fig. 1). Structural comparisons revealed that XGv347 is very similar to A23-58.1, an ultrapotent and broadly reactive neutralizing antibody effective against 23 SARS-CoV-2 variants^22^, but marked differences could be observed in the complementarity-determining region (CDR) domains (Extended Data Fig. 8). Furthermore, the residues of the epitope of XGv347 match with a major subset of those targeted by S2K146, another broadly cross-reactive sarbecovirus neutralizing antibody^23,24^, highlighting a plausible capability of these neutralizing antibodies to cross-neutralize Omicron and circulating SARS-CoV-2 variants. Unexpectedly, the epitopes of XGv347, A23-58.1 as well as their sister neutralizing antibodies would be normally inaccessible for the RBD-down conformation in the WT S, but become accessible for either up or down RBDs in the Omicron S owing to a markedly outward expansion and clockwise rotation of approximately 10º of three RBDs, leading to an approximately 9 Å conformational movement of the receptor-binding motif (RBM) (Fig. 3d, Extended Data Fig. 9). The XGv347 paratope comprised 5 CDRs with heavy chain and light chain contributing 70% and 30% of the binding surface area, respectively (Fig. 3b, c, Extended Data Table 2). Overall XGv289, XGv282 and XGv265 bind patches surrounding the right shoulder of RBD with various orientations^20^, but in a manner similar to those observed for LY-CoV1404, BD-812 and REGN10987—antibodies that are known to generally neutralize most VOCs with high potency^25–27^—but showing decreased, to varying degrees, binding and neutralizing activities against Omicron owing to the presence of new N440K and G446S mutations (Fig. 2b, Extended Data Fig. 10, Extended Data Table 2). Notably, XGv265 and REGN10987 recognize almost the same epitopes, and both have almost no neutralizing activity against Omicron, despite retaining weak binding (Extended Data Fig. 10). Structural superimpositions and competitive BLI assays reveal that XGv347 and either XGv289 or XGv265 can simultaneously bind to S, informing strategies to rationally design two-antibody combinations for potential therapeutics (Extended Data Figs. 11, 12).

## Structural basis for immune escape

**Extended Data Fig. 13:**
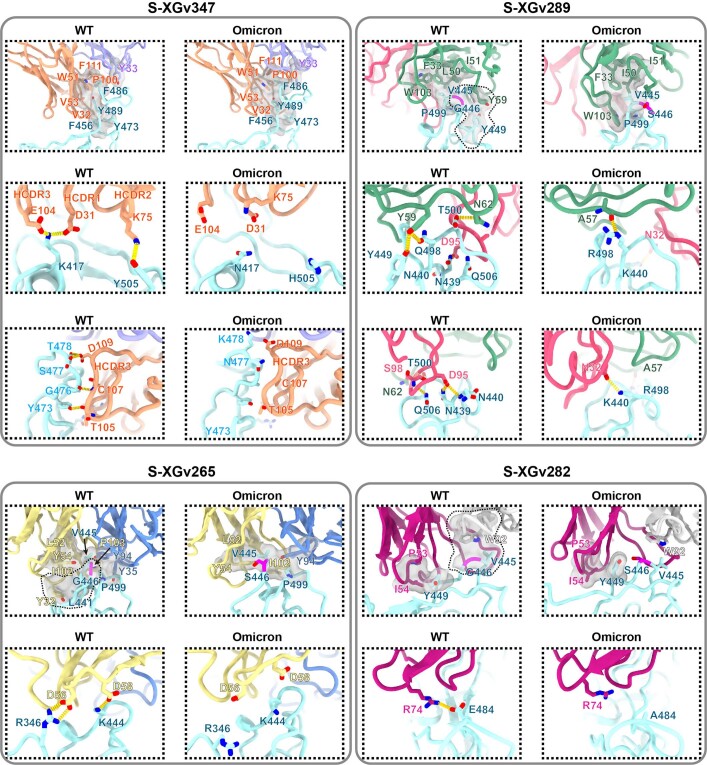
Interactions details between antibodies (XGv347, XGv289, XGv282 and XGv265) and SARS-CoV-2 WT (left) and Omicron RBD (right). All the WT structures are predicted with GROMACS. Hydrophobic patches and hydrogen bonds are denoted by surface and dash lines. Color scheme is the same as in Fig. 3a. For hydrophobic patches of XGv289, XGv282 and XGv265, G446 and S446 are colored in magenta. The dash lines marked out the hydrophobic patches only found in WT RBD.

XGv347, XGv289, XGv282 and XGv265 bound Omicron S with 5- to 40-fold lower affinity compared with their binding to WT S, although the same binding modes were observed for the two orthologues (Fig. 3, Supplementary Table 1). XGv347 exhibited tight binding to WT S primarily owing to extensive hydrophobic interactions contributed by F456, Y473, F486 and Y489 from WT RBD, V32, V53, W51, P100 and F111 from the heavy chain, Y33 from the light chain, and nine hydrogen bonds (Extended Data Fig. 13, Table 3). Hydrophobic interactions between the Omicron RBD and XGv347 were largely maintained. However, substitutions of Y505H and K417N abolished three hydrogen bonds formed with K75, D31 and E104 from the heavy chain complementarity-determining regions (HCDRs), leading to conformational shifts in HCDR3 and the RBM tip (residues 470–490), which further perturbed six hydrogen bonds built by Y473, A475, S477, T478 and Q493 from WT RBD with T105, C107, A56, G55 and D109 from the HCDRs, albeit with an extra hydrogen bond established by the mutation Q493R and G55 from HCDR2 for Omicron (Extended Data Fig. 13). Similarly, a large patch of hydrophobic interactions constructed by V445, G446, Y449 and P499 from the WT RBD and F33, L50, I51, Y59 and W103 from the HCDRs as well as extensive hydrophilic interactions facilitate tight binding between XGv289 and WT S (Fig. 3, Extended Data Fig. 13). Substitution of G446S disrupts the hydrophobic microenvironment, substantially decreasing hydrophobic interactions between Omicron S and XGv289. Furthermore, mutations of N440K and Q498R, together with altered local conformation, also decrease hydrogen bonding formed by N439, K440, Y449, R498, T500 and Q506 from the Omicron RBD and D95, L98 from the light chain complementarity-determining regions (LCDRs) as well as Y59 and N62 from the HCDRs that would exist in the XGv289–WT S complex (Extended Data Fig. 13). Among these four representative antibodies, XGv282 showed a minimal reduction in binding affinity (fivefold), but a more substantial reduction in neutralization (approximately 40-fold), whereas XGv347 showed a 40-fold decrease in binding, but unchanged neutralization against Omicron when compared to WT S (Extended Data Table 3), suggesting that the epitopes, rather than binding affinity, might have more crucial roles in the neutralizing potency and breadth of an antibody. Consistent with XGv289, the substitution of G446S alters the hydrophobic microenvironment generally established by RBD and a group of antibodies bound at the right shoulder, including XGv289 and XGv282, triggering a conformational shift on CDRs and disrupting antibody recognition (Extended Data Fig. 13). In addition, the mutation E484A breaks the hydrogen bond with R74 from XGv282 HCDR2 and losses of charge interactions between R346 and K444 on WT RBD, and D56 and D58 on XGv265 LCDR2 owing to conformational alterations, further decreasing the binding of XGv282 and XGv265 to the Omicron S, respectively (Extended Data Fig. 13). Together, G446S, acting as a critical mutation site, can alter the local conformation at the binding interface, conferring greater resistance to a class of antibodies bound at the right shoulder of the RBD.

## Therapeutic activities of antibodies

**Fig. 4:**
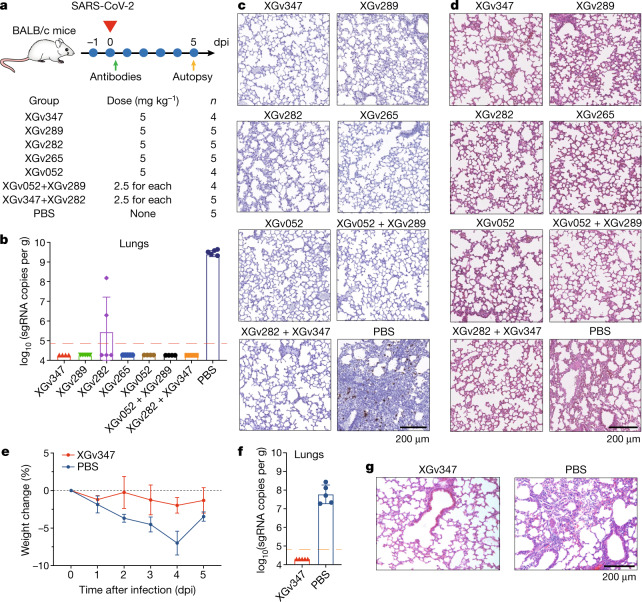
Protection against challenge by SARS-CoV-2 Beta and Omicron variants in mice. **a**, Experimental design for protection assay against Beta variant challenge. *n* = 4 mice in XGv347, XGv052 and XGv052 + XGv289 groups; *n* = 5 mice in other groups. **b**–**d**, Lung tissues of mice challenged with Beta variant, collected at 5 dpi: virus titre (**b**), immunostaining (**c**) and haematoxylin and eosin staining (**d**). **b**, Viral subgenomic (sg) RNA loads in the lungs at 5 dpi were measured by quantitative PCR with reverse transcription. Data are mean ± s.d. Dashed line represents the limit of detection. **c**, In situ hybridization with a SARS-CoV-2 specific probe. Brown staining indicates the presence of SARS-CoV-2 genomic RNA. **d**, Histopathological analysis of lung samples at 5 dpi. **e**, **f**, Weight change (**e**) and viral RNA in lung tissues (**f**) of K18-hACE2 mice challenged with Omicron variant of concern. *n* = 5 mice in each group. **e**, The weight of each mouse in both groups was monitored and recorded daily following infection. Data are mean ± s.d. **f**, Viral RNA loads in the lungs at 5 dpi were measured as in **b**. Data are mean ± s.d. Dashed line represents the limit of detection. **g**, Histopathological analysis of lung tissues from infected mice treated with XGv347 or PBS. Micrographs in **c**, **d**, **g** are representative of two experiments.

**Extended Data Fig. 14:**
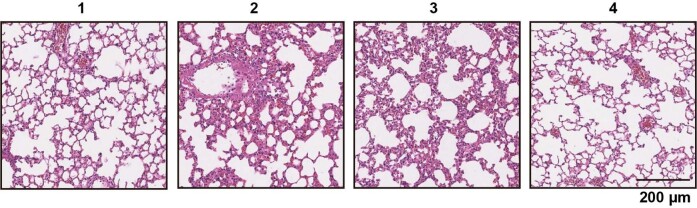
Histopathological analysis of lung samples from XGv282 treatment group at 5 dpi. Shown here are the H&E staining of lung samples from each of the remaining four mice in XGv282 group. Each micrograph is representative of two separate experiments.

Given the excellent neutralizing breadth and potency of these antibodies at the cellular level, we next sought to assess the correlation between in vitro neutralization and in vivo protection. A number of representative monoclonal antibodies with high neutralizing potency and breadth, belonging to different classes, such as XGv347, XGv289, XGv282, XGv265 and XGv052, produced in the HEK 293F cell line were selected for therapeutic evaluation in an established mouse model challenged with Beta virus^28^. Upon intranasal challenge with Beta, adult BALB/c mice showed robust viral replication in the lungs at 3–5 days post inoculation (dpi). To evaluate the protection efficacy of these monoclonal antibodies, BALB/c mice challenged with the Beta variant were administered a single dose of 5 mg kg^−1^ XGv347, XGv289, XGv282, XGv265 and XGv052 individually, or combinations of XGv282 and XGv347 (2.5 mg kg^−1^ of each), and XGv052 and XGv289 (2.5 mg kg^−1^ of each) in therapeutic settings (Fig. 4a). Heavy viral loads with high levels of viral RNA (more than 10^9^ copies per g) were detected in the lungs at day 5 after infection in the control group of mice treated with PBS. However, a single dose of XGv282 reduced the viral RNA loads by about 10,000-fold in the lungs compared with the control group (Fig. 4b). A single dose of XGv289, XGv265, XGv347, XGv052 or antibody cocktails of XGv282 and XGv347 or XGv052 and XGv289 resulted in a complete clearance of viral particles in the lungs (Fig. 4b, c). A potential synergistic effect was observed for combined XGv282 and XGv347 (Fig. 4b, c). In addition, histopathological examination revealed severe interstitial pneumonia, characterized by alveolar septal thickening, inflammatory cell infiltration and distinctive vascular system injury in mice belonging to the control group at day 5 (Fig. 4d). By contrast, no obvious lesions of alveolar epithelial cells or focal haemorrhage were observed in lung sections from mice that received the indicated antibody treatments (Fig. 4d, Extended Data Fig. 14). To further evaluate whether XGv347 could serve as a therapeutic intervention against Omicron in vivo, we tested the protective efficacy of XGv347 on transgenic mice expressing human ACE2^29^ (K18-hACE2) and challenged with Omicron. We recorded the body weight of each mouse daily after infection for 5 days and found that mice in the treatment group maintained their body weight, whereas those in the control group lost weight (Fig. 4e), indicating that XGv347 applied after infection could greatly improve the physiological condition of the Omicron-infected mice. Similar to the studies in mice using Beta, therapeutic administration of XGv347 provided a clear benefit in the K18-hACE2 mice infected with Omicron, as indicated by a complete clearance of viral RNA in the lungs and trachea at day 5 after challenge (Fig. 4f). Of note, K18-hACE2 mice infected with Omicron developed moderate interstitial pneumonia characterized by focal to multifocal widened alveolar interstitium accompanied by infiltration of inflammatory cells (Fig. 4g). No obvious pathological injury was observed in the lung from infected mice treated with XGv347 (Fig. 4g). Collectively, these results suggest that some antibodies—at least best-in-class antibodies such as XGv347—from the repertoire elicited by a three-dose vaccination regimen retain therapeutic potential against currently circulating VOCs.

## Discussion

The ongoing COVID-19 pandemic has resulted in frequent occurrences of SARS-CoV-2 variants that increase transmissibility and reduce potency of vaccine-induced and therapeutic antibodies^4,30^. More recently, there has been concern that the Omicron variant has increased antibody escape breadth owing to newly occurring and accumulated mutations in key epitopes of most neutralizing antibodies. Omicron nearly ablates the neutralization activity of most FDA-approved antibody drugs, including LY-CoV555, LY-CoV016, REGN10933, REGN10987, AZD8895 and AZD1061^2^. These issues mean there is an urgent need to develop new antibody-based therapies that can neutralize these variants as well as future variants of concern. Previous studies revealed that a three-dose vaccination regimen (0, 1 and 7 months) with inactivated SARS-CoV-2 vaccine leads to an improved immune response with significantly enhanced neutralizing breadth via ongoing antibody somatic mutation and memory B cell clonal turnover^7,31^. A subset of highly potent neutralizing antibodies with broad activities (IC_50_ < 0.2 μg ml^−1^) against all circulating VOCs, including Omicron, was present in at least four individuals who had received three doses of inactivated ancestral SARS-CoV-2 vaccine. Some of these antibodies protected against Beta and Omicron infections in mice. Furthermore, our structural and functional analyses revealed that the G446S mutation might act as a critical antibody escape site, conferring greater resistance to one major class of antibodies bound at the right shoulder of RBD by altering microenvironments at the S–neutralizing antibody-binding interface.

In addition to evading currently available antibody therapies, Omicron can diminish the efficacy of clinically approved vaccines, including mRNA and inactivated virus vaccines^30,32^. There is an ongoing debate about whether immune responses can be fine-turned to the Omicron variant by boosting with a tweaked (Omicron-based) vaccine. A major hurdle for this approach is the ‘original antigenic sin’, a phenomenon documented in some other infectious diseases, including flu^33^. The presence of a subset of antibodies with broad neutralizing activities against all circulating VOCs in the memory B-derived antibody repertoire from the three-dose vaccinees suggests a possibility that selective and expeditious recall of humoral responses might be elicited by infection with Omicron or other variants, conferring a secondary protection directed by memory etched in the immune system. Further studies are warranted to examine the advantages and disadvantages of booster shots of an Omicron-specific vaccine or simply administration of a booster with the original vaccines. Last, the identification and characterization of broadly protective antibodies against all circulating VOCs will help in the development of universal vaccination strategies against sarbecoviruses.

## Methods

### Facility and ethics statements

All procedures associated with SARS-CoV-2 live virus were approved by the Animal experiment Committee Laboratory Animal Center, Beijing Institute of Microbiology and Epidemiology with an approval number of IACUC-IME-2021-022 and performed in Biosafety Level 3 (BSL-3) laboratories in strict accordance with the recommendations in the Guide for Care and Use of Laboratory Animals. The procedures about human participants were approved by the Ethics Committee (seal) of Beijing Youan Hospital, Capital Medical University with an approval number of LL-2021-042-K. All participants were provided written informed consent.

### Viral stock and cell lines

SARS-CoV-2 WT strain CN01 was originally isolated from a patient during the early phase of COVID-19 endemic in China. SARS-CoV-2 variant of concern Beta strain GDPCC was isolated in a patient from South Africa and an Omicron strain was isolated from a patient in Hong Kong and now preserved in SinoVac Biotech Ltd. All virus strains were first purified by standard plaque assay as previously described^14^ and then inoculated into Vero cells (ATCC CCL-81) grown to 95% in 10% fetal bovine serum (FBS) supplemented Dulbecco’s minimal essential medium (DMEM) for amplification. Besides, 293T cells (ATCC CRL-3216) and Huh-7 cells (JCRB 0403) were used for pseudovirus neutralization assays; HEK293F cells (Thermo Fisher Scientific 11625019) were used for protein expression; and HEK293 cells (ECACC 85120602) were used for antibody expression. All cells were confirmed to be negative for mycoplasma contamination.

### Human sera samples

The serum samples were obtained from healthy volunteers who had no history of COVID-19 and were verified by PCR and serological assay and received two doses or three doses of CoronaVac (Sinovac) inactivated virus vaccine specific against SARS-COV-2. The whole study was conducted in accordance with the requirements of Good Clinical Practice of China.

### Authentic virus neutralization assay

The serum samples were first incubated at 56 °C for 30 min for inactivation. The heat-treated samples or monoclonal antibodies were subject to seral dilution from 1: 4 or 50 μg ml^−1^ with DMEM in two-fold steps and mixed with a virus suspension containing 100 TCID_50_ at 36. °C for 2 h, after which, the mixtures were added to wells seeded with confluence Vero cells and incubated at 36.5 °C for another 5 days in a humidified 5% CO_2_ cell incubator. After that, the cytopathic effect of each well was observed under microscopes by three different individuals and the related dilutions and concentrations were recorded and used for the titration of samples tested by the method of Reed-Muench^14^.

### Pseudovirus neutralization assay

The pseudotyped viruses bearing the S protein were generated, aliquoted and restored as previously described^18^. In brief, 293T cells were first transfected with the plasmid embedded with the *S* gene of WT or variant (Alpha, Beta, Gamma, Delta, Lambda and Omicron) SARS-CoV-2. The transfected 293T cells were infected with VSV G pseudotyped virus (G*ΔG-VSV) at a multiplicity of infection (MOI) of 4. After incubation for 5 h, cells were washed with PBS, and then complete culture medium was added. After another 24 h, the SARS-CoV-2 pseudoviruses were produced and collected. For the in vitro pseudotyped virus neutralization assay, the plasma samples or antibodies were diluted in DMEM starting from 1:10 or 10 μg ml^−1^ with 6 additional threefold serial dilutions, each of which were mixed with the collected pseudovirus and incubated at 37 °C for 1 h. After that, the mixtures were added to Huh-7 cells and placed back for incubation for another 24 h. Then, the luciferase luminescence (RLU) of each well was measured with a luminescence microplate reader. The neutralization percentage was calculated as following: Inhibition (%) = (1 − (sample RLU − blank RLU)/(positive control RLU − blank RLU)). Antibody neutralization titres were presented as 50% maximal inhibitory concentration (IC_50_).

### Protein expression and purification

The sequences of VOC Omicron full-length S protein (residues 1–1208), RBD (residues 319–541) and NTD (residues 1–304) were modified from the plasmids encoding the S, RBD and NTD of WT SARS-COV-2 (GenBank: MN908947) in our lab by overlapping PCR. In additional to the reported mutations (A67V, Δ69–70, T95I, G142D, Δ143–145, Δ211, L212I, ins214EPE, G339D, S371L, S373P, S375F, K417N, N440K, G446S, S477N, T478K, E484A, Q493R, G496S, Q498R, N501Y, Y505H, T547K, D614G, H655Y, N679K, P681H, N764K, D796Y, N856K, Q954H, N969K and L981F) on Omicron, the proline substitutions at 817, 892, 899, 942, 986 and 987, ‘GSAS’ substitutions at the S1/S2 furin cleavage site (residues 682–685) and a C-terminal T4 foldon trimerization domain were also introduced in the Omicron S construct to stabilize the trimeric conformation of S protein. For protein expression, the plasmids of these proteins were transiently transfected into HEK 293F cells grown in suspension at 37 °C in an incubator supplied with 8% CO_2_, rotating at 130 rpm. The cell supernatants were collected and concentrated three days post-transfection, and further purified by affinity chromatography using resin attached with streptavidin and size-exclusion chromatography (SEC) using a Superose 6 10/300 column (GE Healthcare Life Sciences) equilibrated with the buffer containing 20 mM Tris-HCl, pH 8.0, and 200 mM NaCl.

### Single memory B cell isolation and sequencing

PBMCs were separated from the whole-blood samples obtained from four volunteers using Histopaque (Sigma) gradient centrifugation. After washing with Hank’s balanced salt solution (HBSS) (Solarbio) for three times, the cells were aliquoted and stored in liquid nitrogen in the presence of FBS and DMSO. For single memory B cell sorting, stored PBMCs were thawed and incubated with CD19 MicroBeads (Miltenyi Biotec) to screen out CD19+ B lymphocytes, which were then incubated with human Fc block (BD Biosciences), anti-CD20-PECy7 (BD1113 Biosciences), S-ECD-PE, and S-ECD-APC. The single memory B cells (CD20-1114 PECy7+ S-ECD-PE+ S-ECD-APC+) were further sorted into 96-well plates using a FACSAria II (BD Biosciences), and followed by sequencing and cloning as previously described^35^.

### Antibody expression and Fab generation

The selected 323 antibodies were subjected to gene codon optimization and construction with a plasmid encoding human IgG1 Fc as described previously^7^. Then the clones were transiently transfected into mammalian HEK 293F cells and incubated for 5 days in a 5% CO_2_ rotating incubator at 37 °C for antibody expression, which were further purified using protein A and dialyzed into phosphate buffered saline (PBS). The purified monoclonal antibodies XGv265, XGv282, XGv289 and XGv347 were then processed to obtain their Fab fragments using the Pierce FAB preparation kit (Thermo Scientific) as described previously^36^. In brief, the samples were first applied to desalination columns to remove the salt and the flow-throughs were collected and incubated with papain that was attached with beads to cleave Fab fragments from the whole antibodies for 5 h at 37 °C. After that, the mixtures were transferred into protein A columns and the flow-throughs, that is, the Fab fragments were collected and dialyzed into PBS (ThermoFisher, catalogue (cat.) no. 10010023).

### Bio-layer interferometry

BLI experiments were run on an Octet Red 384 instrument (Fortebio). To measure the binding affinities of monoclonal antibodies, monoclonal antibodies were immobilized onto Protein A biosensors (Fortebio) and threefold serial dilutions of WT RBD, Alpha RBD (ACROBiosystems, cat. no. SPD-C52Hn), Beta RBD (ACROBiosystems, cat. no. SPD-C52Hp), Gamma RBD (ACROBiosystems, cat. no. SPD-C52Hr), Delta RBD (ACROBiosystems, cat. no. SPD-C52Hh) and Omicron RBD (ACROBiosystems, cat. no. SPD-C522e) in PBS were used as analytes. Data were then analysed using software Octet BLI Analysis 12.2 (Fortebio) with a 1:1 fitting model. For the competitive assay by BLI, SARS-CoV-2 WT RBD tagged with His (ACROBiosystems, cat. no. SPD-C52H3) was loaded on NTA biosensors, which were pre-equilibrated in the buffer for at least 1 min. The loaded biosensors were immersed with the first monoclonal antibody for 300 s, followed by addition of the second monoclonal antibody for another 300 s. Data obtained were also analysed by Octet BLI Analsis 12.2.

### ELISA

To evaluate whether the given monoclonal antibodies could block the interaction between human ACE2 (hACE2) and RBD, ACE2 competition ELISA was performed by using the SARS-CoV-2 (B.1.1.529) Inhibitor Screening Kit (ACROBiosystems, cat. no. EP-115) according to the recommended protocol. In brief, each of the 10 twofold dilution series of monoclonal antibodies (starting dilution of 25 μg ml^−1^) and 0.8 μg ml^−1^ of HRP-conjugated SARS-CoV-2 RBD were added into the ELISA plate wells which are pre-coated with hACE2 protein. After incubation at 37 °C for 1 h, the plates were washed three times with PBST (0.1% Tween) and the colorimetric signals were developed by addition of 3,3′,5,5′-tetramethylbenzidine TMB (Thermo Fisher) for 10 min. The reaction was stopped by addition of 50 μl 1 M H_2_SO_4_. The absorbance was measured at 450 nm with an ELISA microplate reader. For each monoclonal antibody, a blank control with no monoclonal antibody was added for inhibition calculation. The area under the curve (AUC) of each monoclonal antibody were determined using Prism V8.0 (GraphPad). For competitive ELISAs to identify the domain of a given monoclonal antibody, 96-well plates were first coated with RBD (2 μg ml^−1^) and then blocked with 2% BSA in PBS. After incubation with the reference monoclonal antibodies, the blocking antibody (15 μg ml^−1^), the wells were followed by directly adding the second biotinylated antibodies (0.25 μg ml^−1^). Streptavidin-HRP (BD Biosciences) was then added for detection. Samples with no first antibody were used as a negative control for normalization.

### Cryo-EM sample preparation, data collection

The purified S protein was mixed with each of the Fab fragments of XGv265, XGv282, XGv289 or XGv347 with a molar ratio of 1: 1.2 for 10 s ice incubation, and then dropped onto the pre-glow-discharged holey carbon-coated gold grid (C-flat, 300-mesh, 1.2/1.3, Protochips In.), blotted for 7 s with no force in 100% relative humidity and immediately plunged into the liquid ethane using Vitrobot (FEI). Cryo-EM data sets of these complexes were collected at 300 kV with an FEI Titan Krios microscope (FEI). Movies (32 frames, each 0.2 s, total dose of 60 e^−^ Å^−2^) were recorded using a K3 Summit direct detector with a defocus range between 1.5–2.7 μm. Automated single particle data acquisition was carried out by SerialEM, with a calibrated magnification of 22,500 yielding a final pixel size of 1.07 Å.

### Cryo-EM data processing

A total of 3,752, 2,631, 3,955 and 5,014 micrographs of S–XGv265 complex, S–XGv282 complex, S–XGv289 complex and S–XGv347 complex, respectively were recorded and subjected to beam-induced motion correction using motionCorr in Relion 3.0 package^37^. The defocus value of each image was calculated by Gctf. Then, 1,302,103, 756,508, 2,332,045 and 2,320,416 particles of the S–XGv265 complex, S–XGv282 complex, S–XGv289 complex and S–XGv347 complex, respectively, were picked and extracted for reference-free 2D alignment by cryoSPARC^38^, based of which, 422,083, 190,154, 837,832 and 614,852 particles were selected and applied for 3D classification by Relion3.0 for S–XGv265 complex, S–XGv282 complex, S–XGv289 complex and S–XGv347 complex, respectively with no symmetry imposed to produce the potential conformations for the complexes. Afterwards, the candidate model for each complex was selected and processed by non-uniform auto-refinement and postprocessing in cryoSPARC to generate the final cryo-EM density for S–XGv265 complex, S–XGv282 complex, S–XGv289 complex and S–XGv347 complex. To improve the resolution of the interface between RBD and monoclonal antibodies, the block-based reconstruction was performed to obtain the final resolution of the focused interfaces which contained the interfaces of RBD and monoclonal antibodies investigated here as described previously^39^. The resolution of each structure was determined on the basis of the gold-standard Fourier shell correlation (threshold = 0.143) and evaluated by ResMap. All dataset processing is shown in Extended Data Fig. 3 and summarized in Extended Data Table 2.

### Model fitting and refinement

The atomic models of the complexes were generated by first fitting the chains of the native apo SARS-CoV-2 S trimer (PDB number of 6VYB) and Fabs (PDB number of 7LSS and 7CZW for XGv265, 5MES and 5VAG for XGv282, 6UDA and 7MEG for XGv289 as well as 7E3K for XGv347) into the cryo-EM densities of the final S-Fab-complexes described above by Chimera, followed by manually adjustment and correction according to the protein sequences and densities in Coot, as well as real space refinement using Phenix. Details of the refinement statistics of the complexes are summarized in Extended Data Table 2.

### Molecular dynamics simulation and Δ*G* estimation

Model of SARS-CoV-2 WT RBD in complex with XGv265, XGv282, XGv289 and XGv347 were generated in Chimera by superimposition of WT RBD and cryo-EM structure of Omicron RBD in complex with the four antibodies. Before molecular dynamics, all models were checked by WHAT IF Web Interface (https://swift.cmbi.umcn.nl/servers/html/index.html) to model missing sidechains and remove atomic clashes. After that, the structure was simulated by GROMACS-2021. In brief, we used OPLS force field with TIP3P water model to prepare the dynamic system and add Na+ and Cl^-^ ions to make the system electrically neutralized. Then, the system was subjected to energy minimization using the steepest descent algorithm until the maximum force of 1,000 kJ mol-1 has been achieved. NVT ensemb1e via the Nose-Hoover method at 300 K and NPT ensemble at 1 bar with the Parinello-Rahman algorithm were employed successively to make the temperature and the pressure equilibrated, respectively. Finally, molecular dynamics production runs of 100 ns were performed starting from random initial velocities and applying periodic boundary conditions. The non-bonded interactions were treated using Verlet cut-off scheme, while the long-range electrostatic interactions were treated using particle mesh Ewald method. The short-range electrostatic and van der Waals interactions were calculated with a cut-off of 12 Å. Average structure of the four complexes were generated using the last 10 ns frames and Δ*G* between the antibodies and RBD was estimated in ROSETTA by InterfaceAnalyzer. Atomic_burial_cutoff, sasa_calculator_probe_radius and interfaces_cutoff values were set to 0.01, 1.4 and 8.0 respectively.

### In vivo protection against SARS-CoV-2 Beta and Omicron variants challenge in mice

The in vivo protection efficacies of single antibody or antibody cocktails were assessed by using a newly established mouse model based on a SARS-CoV-2 Beta variant strain^28^. In brief, groups of 8-month-old female BALB/c mice were infected with 1 × 10^4^ PFU of SARS-CoV-2 Beta variant strain, then infected mice were treated intraperitoneally with a single dose of different antibodies or antibody cocktails (5 mg kg^−1^) at 1 h after infection. The protection efficacy of XGv347 was also assessed by using 10-week-old female K18-hACE2 mice, each challenged with 1 × 10^2^ TCID_50_ of Omicron strain. And two 2 h post infection, mice were intraperitoneally treated with a single dose of XGv347 at 30 mg kg^−1^ or the same volume of PBS as control. The lung tissues of mice from both two groups were collected at 5 dpi for viral RNA loads assay and pathological examination. All mice were randomly allocated in each group; investigators were not blinded to allocation during the experiment and outcome assessment.

### Viral burden determination

Viral burden in lung from mice were measured as described previously^17^. In brief, lung tissue homogenates were clarified by centrifugation and viral RNA was extracted using the QIAamp Viral RNA Mini Kit (Qiagen). Viral sgRNA quantification in each tissue sample was performed by quantitative reverse transcription PCR (RT-qPCR) targeting the S gene of SARS-CoV-2. RT-qPCR was performed using One-Step PrimeScript RT-PCR Kit (Takara).

### Histology and RNA in situ hybridization

Lung tissues from mice were fixed with perfusion fixative (formaldehyde) for 48 h, and embedded in paraffin according to standard histological assays. For histopathology, lung tissues were stained with haematoxylin and eosin. Images were captured using Olympus BX51 microscope equipped with a DP72 camera. For RNA ISH assays were performed with an RNAscope 2.5 (Advanced Cell Diagnostics) according to the manufacturer’s instruction. In brief, formalin-fixed paraffin-embedded tissue sections of 5 μm were deparaffinized by incubation for 60 min at 60 °C. Endogenous peroxidases were quenched with hydrogen peroxide for 10 min at room temperature. Slides were then boiled for 15 min in RNAscope Target Retrieval Reagents and incubated for 30 min in RNAscope Protease Plus before probe hybridization. The probe targeting 2019-nCoV RNA was designed and synthesized by Advanced Cell Diagnostics (cat. no. 848561). Tissues were counterstained with Gill’s haematoxylin and visualized with standard bright-field microscopy. Original magnification was 10×.

### Reporting summary

Further information on research design is available in the Nature Research Reporting Summary linked to this paper.

## Online content

Any methods, additional references, Nature Research reporting summaries, source data, extended data, supplementary information, acknowledgements, peer review information; details of author contributions and competing interests; and statements of data and code availability are available at 10.1038/s41586-022-04466-x.

### Supplementary information

Reporting Summary

Supplementary Table 1 Information for the antibodies isolated individuals who received 3 doses of Coronavac vaccine.

Supplementary Table 2 Sequences of the 41 representative recombinant antibodies.

## Figures and Tables

**Extended Data Table 1:**
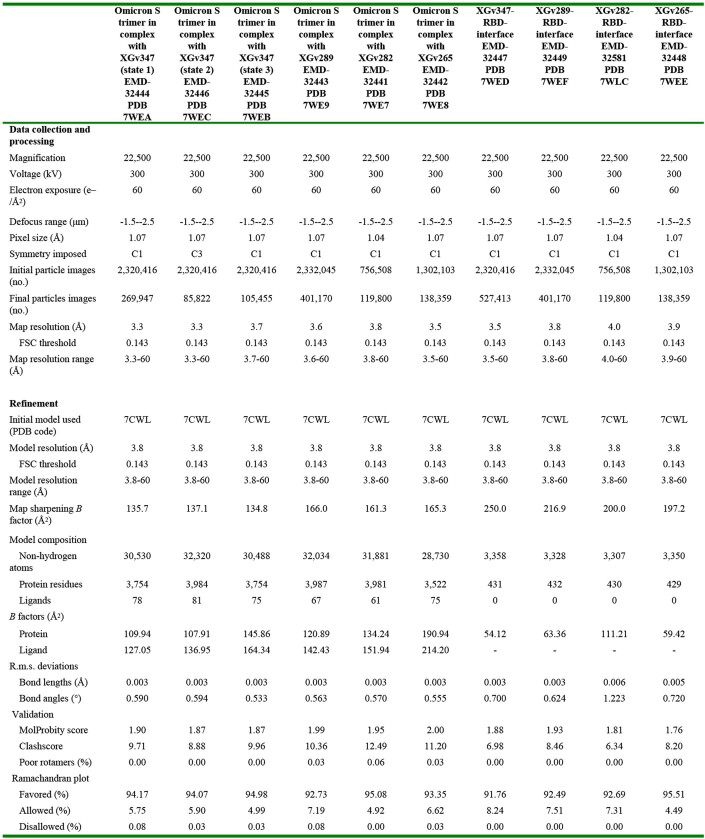
Statistics for cryo-EM data collection, refinement, and validation

**Extended Data Table 2:**
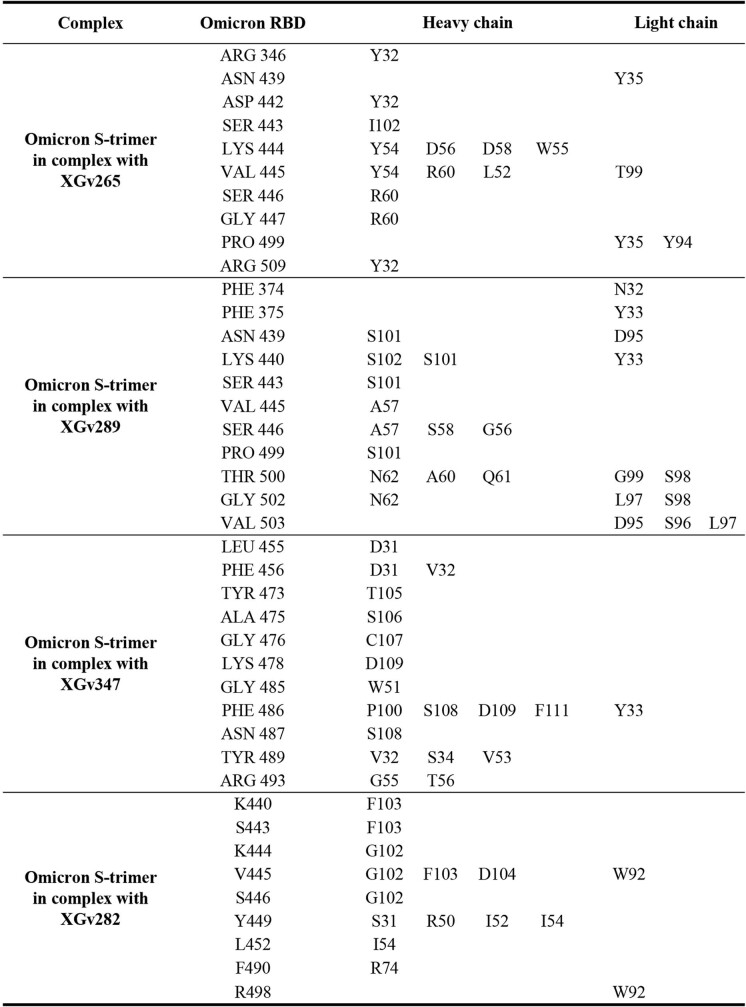
List of interacting residues between Fabs and Omicron SARS-CoV-2 S trimer (d < 4 Å)

**Extended Data Table 3:**
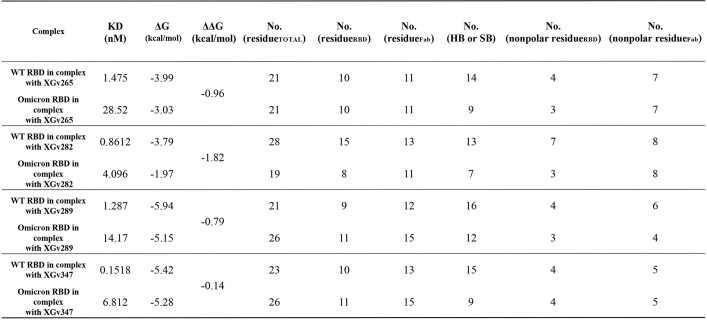
Statistics for molecular dynamics

HB, hydrogen bond; SB, salt bridge.

## Data Availability

The atomic coordinates of XGv347 in complex with S trimer (state 1), XGv347 in complex with S trimer (state 2) and XGv347 in complex with S trimer (state 3) have been submitted to the Protein Data Bank with accession codes 7WEA, 7WEC and 7WEB, respectively. The atomic coordinates of XGv265, XGv282 and XGv289 have been deposited in the Protein Data Bank under accession codes 7WE8, 7WE7 and 7WE9, respectively. Cryo-EM density maps in this study have been deposited at the Electron Microscopy Data Bank with accession codes EMD-32444 (state 1), EMD-32446 (state 2) and EMD-32445 (state 3), EMD-32441 (XGv282), EMD-32442 (XGv265) and EMD-32443 (XGv289). To reveal structural details of Fab binding mechanism, the local optimized method was used to optimized data progress and the related atomic models and EM density maps of optimized reconstructions of Fab interaction interfaces have been deposited under accession codes 7WEE (XGv265), 7WED (XGv347), 7WLC (XGv282), 7WEF (XGv289), EMD-32447 (XGv347), EMD-32448 (XGv265), EMD-32581 (XGv282) and EMD-32449 (XGv289).
