# Supplementary material for: Memory B cell repertoire from triple vaccinees against diverse SARS-CoV-2 variants
Source: Nature. 2022 Jan 28;603(7903):919–25. doi: 10.1038/s41586-022-04466-x (PMC8967717; doi:10.1038/s41586-022-04466-x)
Supplement: Supplementary file 1 — Reporting Summary [file 41586_2022_4466_MOESM1_ESM.pdf]

nature portfolio

Corresponding author(s): Xiangxi Wang

Last updated by author(s): Jan 12, 2022

Reporting Summary

Nature Portfolio wishes to improve the reproducibility of the work that we publish. This form provides structure for consistency and transparency in reporting. For further information on Nature Portfolio policies, see our Editorial Policies and the Editorial Policy Checklist.

Statistics

For all statistical analyses, confirm that the following items are present in the figure legend, table legend, main text, or Methods section.

n/a	Confirmed	
☐	☒	The exact sample size (*n*) for each experimental group/condition, given as a discrete number and unit of measurement
☐	☒	A statement on whether measurements were taken from distinct samples or whether the same sample was measured repeatedly
☒	☐	The statistical test(s) used AND whether they are one- or two-sided<br>*Only common tests should be described solely by name; describe more complex techniques in the Methods section.*
☒	☐	A description of all covariates tested
☒	☐	A description of any assumptions or corrections, such as tests of normality and adjustment for multiple comparisons
☐	☒	A full description of the statistical parameters including central tendency (e.g. means) or other basic estimates (e.g. regression coefficient) AND variation (e.g. standard deviation) or associated estimates of uncertainty (e.g. confidence intervals)
☒	☐	For null hypothesis testing, the test statistic (e.g. *F*, *t*, *r*) with confidence intervals, effect sizes, degrees of freedom and *P* value noted<br>*Give P values as exact values whenever suitable.*
☒	☐	For Bayesian analysis, information on the choice of priors and Markov chain Monte Carlo settings
☒	☐	For hierarchical and complex designs, identification of the appropriate level for tests and full reporting of outcomes
☒	☐	Estimates of effect sizes (e.g. Cohen's *d*, Pearson's *r*), indicating how they were calculated

Our web collection on statistics for biologists contains articles on many of the points above.

Software and code

Policy information about availability of computer code

Data collection	Serial EM3.8
Data analysis	GROMACS2021,RELION 3.0, cryoSPARC 3.3.1, Chimera 1,15, Chimerax 1.1, coot0.9.4, Phenix 1.19 and GraphPad Prism 9.2.0 were used for data analysis.

For manuscripts utilizing custom algorithms or software that are central to the research but not yet described in published literature, software must be made available to editors and reviewers. We strongly encourage code deposition in a community repository (e.g. GitHub). See the Nature Portfolio guidelines for submitting code & software for further information.

Data

Policy information about availability of data

All manuscripts must include a data availability statement. This statement should provide the following information, where applicable:

- Accession codes, unique identifiers, or web links for publicly available datasets
- A description of any restrictions on data availability
- For clinical datasets or third party data, please ensure that the statement adheres to our policy

Data availabilityThe atomic coordinates of XGv347 in complex with S trimer (state I), XGv347 in complex with S trimer (state II), XGv347 in complex with S trimer (state III), XGv347-S have been submitted to the Protein Data Bank with accession numbers: 7WEA, 7WEC and 7WEB, respectively. Futhermore, the atomic coordinates of XGv265, XGv282 and XGv289 have been deposited in the protein data bank under accession code 7WE8, 7WE7 and 7WE9, respectively. Cryo-EM density maps in this study have been deposited at the Electron Microscopy Data Bank with accession codes EMD-32444 (state1), EMD-32446 (state2) and EMD-32445 (state3), EMD-32441 (XGv282), EMD-32442 (XGv265), and EMD-32443 (XGv289). To reveal structural details of Fab binding mechanism, the local optimized method are used to optimized data progress and the related atomic models and EM density maps of optimized reconstructions of Fab interaction

interface has been deposited under accession code 7WEE (XGv265), 7WED (XGv347), 7WLC (XGv282), 7WEF (XGv289), EMD-32447 (XGv347), EMD-32448 (XGv265), EMD-32581(XGv282), EMD-32449 (XGv289), respectively.

Field-specific reporting

Please select the one below that is the best fit for your research. If you are not sure, read the appropriate sections before making your selection.

☒ Life sciences ☐ Behavioural & social sciences ☐ Ecological, evolutionary & environmental sciences

For a reference copy of the document with all sections, see nature.com/documents/nr-reporting-summary-flat.pdf

Life sciences study design

All studies must disclose on these points even when the disclosure is negative.

Sample size	No statistical methods were used to predetermine sample size. We obtained 120 human serums, all the vaccine sera were collected from volunteers who received two doses or three doses of the WHO-approved inactivated SARS-CoV-2 vaccine (CorovaVac, Sinovac, China). For the animal study, 37 BALB/c mice and 10 K18-hACE2 mice were used for protection experiments.
Data exclusions	No data excluded.
Replication	All experiments were performed and verified in multiple replicates as indicated in their methods/figure legends.
Randomization	All mice were divided into the given groups (7 for Beta strain challenge and 2 for Omicron strain challenge) randomly.
Blinding	Volunteers received vaccinations open-label. The investigators were not blinded to allocation during experiments and outcome assessment. Data collection and analysis were performed by different people, the sample classification were replaced by simple marks during data analysis

Reporting for specific materials, systems and methods

We require information from authors about some types of materials, experimental systems and methods used in many studies. Here, indicate whether each material, system or method listed is relevant to your study. If you are not sure if a list item applies to your research, read the appropriate section before selecting a response.

Materials & experimental systems

n/a	Involved in the study	
☐	☒	Antibodies
☐	☒	Eukaryotic cell lines
☒	☐	Palaeontology and archaeology
☐	☒	Animals and other organisms
☐	☒	Human research participants
☒	☐	Clinical data
☒	☐	Dual use research of concern

Methods

n/a	Involved in the study	
☒	☐	ChIP-seq
☒	☐	Flow cytometry
☒	☐	MRI-based neuroimaging

Antibodies

Antibodies used	XGv347,XGv286,XGv051,XGv303,XGv264,XGv293,XGv052,XGv337,XGv338,XGv261,XGv302,XGv289,XGv291,XGv042,XGv297,XGv288, XGv055,XGv295,XGv345,XGv074,XGv016,XGv292,XGv290,XGv304,XGv031,XGv282,XGv053,XGv387,XGv296,XGv253,XGv287,XGv294, XGv050,XGv017,XGv177,XGv026,XGv265,XGv420,XGv402,XGv285,XGv266, VIR-7831, DXP-604, AZD8895, REGN10987, LY-CoV016
Validation	All of the XGv series SARS-CoV-2 spike antigen-specific monoclonal antibodies have been validated for use in ELISA, BLI and neutralizing SARS-CoV-2 pseudovirus/authentic virus first time in this study. S309, VIR-7831, DXP-604, AZD8895, REGN10987, LY-CoV016 have been validated in previous publications cited in this paper. Specifically,VIR-7831 was tested in Pinto, D et al 2020, Nature;DXP-604 was tested in Shuo, D. et al 2020, Cell; AZD8895 was tested in Jinhui,D. et al 2021,Nat Microbiol; REGN10987 was tested in Johanna Hansen et al 2020, Science;LY-CoV016 was tested in Shi, R. et al 2020, Nature.

Eukaryotic cell lines

Policy information about cell lines

Cell line source(s)	HEK293T cells (ATCC, cat. no. CRL-3216), Huh-7 cells (Japanese Collection of Research Bioresources [JCRB], cat. no. 0403),HEK293F cells (Thermo Fisher, cat. no. 11625019)
Authentication	The authentication of cells have been confirmed using STR method

Mycoplasma contamination	These cell lines tested negative for mycoplasma.
Commonly misidentified lines (See ICLAC register)	None

Animals and other organisms

Policy information about studies involving animals; ARRIVE guidelines recommended for reporting animal research

Laboratory animals	Groups of BALB/c and K18-hACE2 mice were used. All mice were group-housed conventionally on a 12-h light/dark cycle for 3 days before any experiments,the environmental conditions were maintained thermostatically between 18°C-23°C with 40%-60% humidity.
Wild animals	No wild animals were used in the study
Field-collected samples	No field collected samples were used in the study.
Ethics oversight	All procedures associated with SARS-CoV-2 live virus were approved by the Animal experiment Committee Laboratory Animal Center, Beijing Institute of Microbiology and Epidemiology with an approval number of IACUC-IME-2021-022 and performed in Biosafety Level 3 (BSL-3) laboratories in strict accordance with the recommendations in the Guide for Care and Use of Laboratory Animals.

Note that full information on the approval of the study protocol must also be provided in the manuscript.

Human research participants

Policy information about studies involving human research participants

Population characteristics	Plasma samples were obtained from volunteers who received two doses or three doses of the WHO-approved inactivated SARS-COV-2 vaccine (CorovaVac, Sinovac, China).Median age of participants was 37 years. 44% of volunteers were males and 56% were females.
Recruitment	All the volunteers were recruited by Sinovac, Inc. None of the volunteers had a history of prior SARS-CoV-2 infection and none reported serious adverse events after vaccination.
Ethics oversight	The procedures about human participants were approved by the Ethics Committee (seal) of Beijing Youan Hospital, Capital Medical University with an approval number of LL-2021-042-K. All participants provided written informed consent.

Note that full information on the approval of the study protocol must also be provided in the manuscript.
